# Hot Electron-Driven Photocatalysis Using Sub-5 nm Gap Plasmonic Nanofinger Arrays

**DOI:** 10.3390/nano12213730

**Published:** 2022-10-24

**Authors:** Yunxiang Wang, Buyun Chen, Deming Meng, Boxiang Song, Zerui Liu, Pan Hu, Hao Yang, Tse-Hsien Ou, Fanxin Liu, Halton Pi, Irene Pi, Isleen Pi, Wei Wu

**Affiliations:** 1 Ming Hsieh Department of Electrical and Computer Engineering, University of Southern California, Los Angeles, CA 90089, USA; 2 Wuhan National Laboratory for Optoelectronics, Huazhong University of Science and Technology, Wuhan 430074, China; 3 Department of Applied Physics, Zhejiang University of Technology, Hangzhou 310023, China; 4 Department of Biophysics, Johns Hopkins University, Baltimore, MD 21218, USA; 5 School of Computer Science, Carnegie Mellon University, Pittsburgh, PA 15213, USA; 6 College of Art and Science, Washington University in St. Louis, St. Louis, MO 63130, USA

**Keywords:** hot electron, photocatalysis, plasmonic, nanofinger

## Abstract

Semiconductor photocatalysis has received increasing attention because of its potential to address problems related to the energy crisis and environmental issues. However, conventional semiconductor photocatalysts, such as TiO_2_ and ZnO, can only be activated by ultraviolet light due to their wide band gap. To extend the light absorption into the visible range, the localized surface plasmon resonance (LSPR) effect of noble metal nanoparticles (NPs) has been widely used. Noble metal NPs can couple incident visible light energy to strong LSPR, and the nonradiative decay of LSPR generates nonthermal hot carriers that can be injected into adjacent semiconductor material to enhance its photocatalytic activity. Here we demonstrate that nanoimprint-defined gap plasmonic nanofinger arrays can function as visible light-driven plasmonic photocatalysts. The sub-5 nm gaps between pairs of collapsed nanofingers can support ultra-strong plasmon resonance and thus boost the population of hot carriers. The semiconductor material is exactly placed at the hot spots, providing an efficient pathway for hot carrier injection from plasmonic metal to catalytic materials. This nanostructure thus exhibits high plasmon-enhanced photocatalytic activity under visible light. The hot carrier injection mechanism of this platform was systematically investigated. The plasmonic enhancement factor was calculated using the finite-difference time-domain (FDTD) method and was consistent with the measured improvement of the photocatalytic activity. This platform, benefiting from the precise controllable geometry, provides a deeper understanding of the mechanism of plasmonic photocatalysis.

## 1. Introduction

Photocatalysis, a process that can convert solar energy into chemical energy, has garnered great interest for decades due to its great potential for environmental and energy applications [1,2,3,4,5,6]. Semiconductor photocatalysts play an important role in photocatalysis due to their unique chemical and physical properties. However, the wide band gap of these materials has limited their photocatalytic efficiency under solar light. For instance, the band gap of TiO_2_ is 3.2 eV, which means that only ultraviolet light, which accounts for ~4% of the solar energy, can be harvested to accelerate the chemical reaction. Their poor ability to utilize visible light energy is an obstacle to the further improvement of their performance. Conversely, noble metal NPs such as Au and Ag strongly interact with visible light. The nanoparticles support surface plasmon resonances with optical cross-sections far larger than their physical cross-sections and produce strong electric field near the surface [7]. The generated energetic hot carriers, due to the surface plasmon decays, can then directly drive chemical reactions [8,9,10]. However, the plasmon-induced hot carriers are rapidly recombined and deactivated on the timescale of femtoseconds [11]. The short lifetime of hot carriers limits the charge utilization efficiency in many photocatalysis reactions. Integrating noble metal NPs with traditional semiconductor photocatalysts is a practical and highly general approach for addressing this problem [12,13,14]. The hot carriers can be injected into the adjacent semiconductors at the heterojunction to achieve a larger charge separation and thus longer lifetimes [15,16]. This type of visible light-driven plasmonic photocatalyst has shown high efficiency in various applications, such as water splitting [17,18,19], CO_2_ reduction [13,20,21] and dye degradation [22,23,24,25].

The long-standing challenge in such plasmonic photocatalysts is determining how the plasmonic energy can be efficiently utilized. An ideal plasmonic photocatalyst should have two properties: (1) to strongly interact with visible light to produce strong LSPR; (2) to show a high transfer efficiency for the energy stored in the surface plasmon. Extensive studies have shown that noble metal NP pairs with subnanometer gaps can support ultra-strong LSPR at the hot spot between the two particles [26,27,28,29,30]. A straightforward way to utilize this enhancement is to place semiconductor materials exactly at the hot spots, providing an efficient pathway for the harvested energy to be dissipated by forming hot carriers. As molecules can be trapped inside the hot spot [31], the generated hot carriers can transfer to the molecules and efficiently participate in chemical reactions. While many attempts have been made to fabricate plasmonic nanostructures with subnanometer gaps [32,33,34,35], placing semiconductor materials right at the high spots is still challenging. According to our previous study, collapsible nanofingers are a great platform to realize such plasmonic photocatalysts [26,27,28]. Collapsible nanofingers are a type of three-dimensional nanostructure where TiO_2_-coated Au nanoparticles are placed on the top of high-aspect-ratio flexible polymer pillars. The adjacent nanofingers can collapse under the capillary force of the ethanol solution, and the Au nanoparticles with a TiO_2_ coating touch each other, forming hot spots at the gaps. The gap size is well-defined by twice the thickness of the TiO_2_ layer. As the plasmonic resonance can be controlled by tuning the gap size between the two Au nanoparticles [36,37,38], we can achieve high-electric field enhancement under visible light. In addition, the TiO_2_ thin film is placed exactly at the hot spots, ensuring most of the harvested energy can be utilized. In this work, the photocatalytic activity of this plasmonic photocatalyst under visible light exposure was evaluated using the photo-degradation of methyl orange (MO). The concentration of MO at different reaction times was measured using UV-Vis spectroscopy. In the control experiments, the TiO_2_ thin film and uncollapsed nanofingers were used to drive the reaction under the same conditions. Compared to the TiO_2_ thin film, the uncollapsed nanofingers and collapsed nanofingers showed a 3.5× and 18.5× photocatalytic enhancement, respectively. The local field enhancement provided by the AuNP, which was responsible for the photocatalytic enhancement, was further verified using finite-difference time-domain (FDTD) simulations.

## 2. Materials and Methods

### 2.1. Fabrication of the Device

The fabrication process is shown in Figure S1 in the Supplementary Information. The fabrication of collapsible nanofingers was based on nanoimprint lithography (NIL), reactive-ion etching (RIE) and atomic layer deposition (ALD). A 600 nm UV nanoimprint resist (I-UVP 15% concentration, EZImprinting Inc., Los Angeles, CA, US) was spin-coated onto a 3-inch silicon substrate at 2000 rpm for 10 s, followed by curing under 4 mW/cm^2^ i-line (365 nm) UV exposure for 5 min. A 100 nm lift-off underlayer (IULP 3.5% concentration, EZImprinting Inc.) was spin-coated at 4000 rpm for 40 s onto the UV nanoimprint resist, followed by baking at 120 °C for 5 min. Another 100 nm UV nanoimprint resist (I-UVP 4.1% concentration, EZImprinting Inc.) layer was spin-coated at 2500 rpm for 10 s onto the lift-off underlayer. NIL was performed using a two-dimensional grid mold that was prepared by self-developed interference lithography to form two-dimensional hole array on the thin UV nanoimprint resist layer. Residual layer and the underlying lift-off underlayer were then removed with RIE (Oxford PlasmaPro 100, Oxford Instruments, Concord, MA, USA) to expose the thick UV nanoimprint resist layer. Then, 50 nm Au was evaporated at a normal incidence onto the sample, followed by lift-off process using a hot acetone bath to form Au nanoparticle array onto the thick UV imprint resist layer. UV nanoimprint resist under Au array was subsequently etched by RIE to obtain Au-capped high-aspect-ratio polymer nanofingers. A 2 nm TiO_2_ film was deposited on the nanofingers using a plasma-enhanced ALD (Ultratech Simply ALD, Veeco, Plainview, NY, USA). Finally, the fabricated nanofingers were soaked into ethanol and then air-dried at room temperature. Under the action of capillary force from the ethanol solution, four adjacent nanofingers gradually approached and touched each other, forming AuNP pairs on the top of nanofingers. Van der Waals forces can keep these nanofingers from separating once they touch [39]. In contrast to the nanofinger samples, the control samples should only have 2 nm TiO_2_ film as the photocatalyst and maintain the same area as the nanofinger samples. As the AuNPs also serve as the etching mask in the fabrication process, the nanofinger array cannot be obtained without AuNPs. Thus, the control samples were prepared using ALD to deposit 2 nm TiO_2_ film on silicon substrates with same area of the nanofinger samples.

### 2.2. Characterization

Scanning electron microscopy (SEM) images of the nanofingers before and after collapsing process were taken using JEOL JSM 7001 (JEOL Ltd., Tokyo, Japan). All images were acquired at 8 kV. To demonstrate the coverage of the TiO_2_ film on the AuNPs, transmission electron microscopy (TEM) and energy-dispersive X-ray spectroscopy (EDS) cross-sectional analysis of the nanogaps was performed. The samples for TEM characterization were first prepared using dual beam FIB (Seiko 4050MS, Seiko Instruments Inc., Tokyo, Japan), and then TEM cross-section characterization was performed using JEOL JEM 2100F (JEOL Ltd., Tokyo, Japan).

### 2.3. Electromagnetic Field Simulation

Finite-difference time-domain (FDTD) method was performed to simulate the electromagnetic field distribution near the nanofingers. The nanofinger was modeled by a polymer cylinder that was capped on top by a 50 nm thick Au nanoparticle and coated with 2 nm TiO_2_ film. The diameter and height of the cylinder was 60 nm and 350 nm, respectively. The refractive index of TiO_2_ was derived using an ellipsometer (VAS Ellipsometer, J.A. Woollam, Lincoln, NE, USA), and the refractive index of Au was obtained from the material database in the FDTD software. The refractive index of the polymer was set as 1.48 to approximate UV nanoimprint resist. The nanofinger was placed on top of an infinite substrate and excited with a 532 nm plane wave at normal incidence.

### 2.4. Photocatalysis Measurements

After fabrication, the nanofinger samples and controls samples were cut into 1 cm^2^ square pieces for photocatalysis measurement. One piece of sample and 1 mL MO solution (20 mg/L) were added into a customized transparent reaction vessel (VWR). The solution was stirred by a magnetic stirrer bar at 500 rpm/min. A high-power optically pumped semiconductor laser (COHERENT Verdi G5 SLM, Santa Clara, CA, USA, 532 nm) was used as the light source. The light spot on the sample was 25 mm^2^. A UV-VIS-NIR spectrophotometer (PerkinElmer Lambda 950, PerkinElmer, Waltham, MA, USA) was used to analyze the concentration of methyl orange at different reaction times.

## 3. Results and Discussion

**Figure 1:**
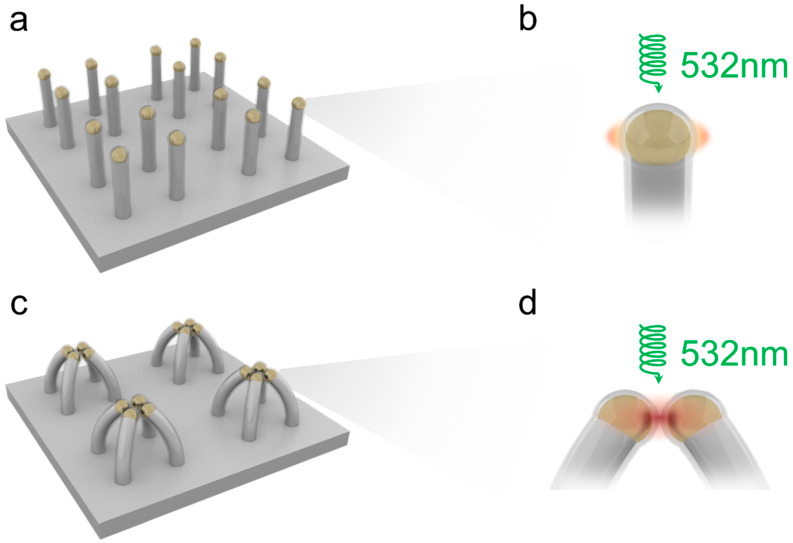
(**a**,**c**) Schematic diagram of collapsible nanofingers before and after collapsing process. (**b**,**d**) Schematic of the electric field enhancement of single nanofinger and collapsed nanofingers.

**Figure 2:**
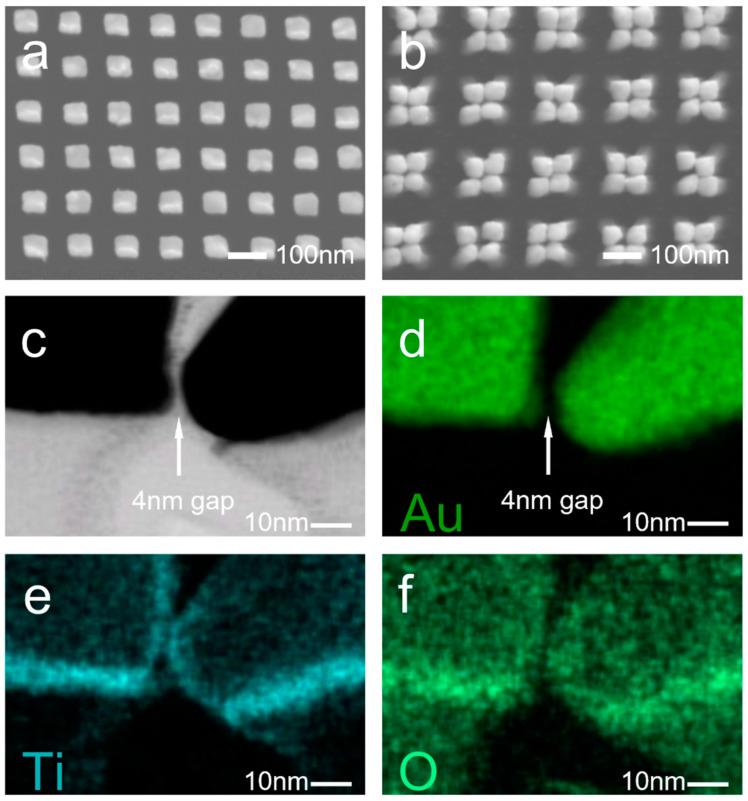
(**a**) SEM image of nanofingers before collapse. (**b**) SEM image of nanofingers after collapse. (**c**) TEM image of the dielectric nanogap between the collapsed nanofingers. (**d**) EDS mapping of Au in the TEM image. (**e**) EDS mapping of Ti in the TEM image. (**f**) EDS mapping of O in the TEM image.

We invented and demonstrated a technique to fabricate a large-area gap plasmon photocatalyst with high reliability and repeatability by combing collapsible nanofingers with a thin dielectric film deposition. Figure 1a,c show the schematic illustration of the nanofingers before and after the collapsing process. The Au nanoparticles were deposited on the top of the high-aspect-ratio UV nanoimprint resist pillars. A thin layer of TiO_2_ was uniformly coated on the nanoparticles using ALD before the collapsing process. The diameter and height of each nanofinger was 60 nm and 350 nm, respectively, and the pitch was 200 nm. As the ALD process deposits the dielectric films with a high conformity and atomic precision [40], the gap size between the two AuNPs was accurately defined by twice the TiO_2_ film thickness. To obtain the strongest field enhancement at the hot spots, the plasmonic properties can be optimized by tuning the thickness of the TiO_2_ film which imposes different tunneling barrier heights for the electrons [41,42,43]. According to our previous work, a 4 nm gap size can provide the strongest field enhancement at the gaps [26]. Figure 1b,d exhibit the electric field enhancement of the uncollapsed and collapsed nanofingers, respectively. The gap plasmon produced by the collapsed nanofingers can produce a much higher electric field enhancement compared to the surface plasmon on the single nanoparticles, and thus the collapsed nanofingers can exhibit higher photocatalytic activity under visible light. The fabricated nanofingers were investigated using SEM, TEM and EDS methods, and the results are shown in Figure 2. Figure 2a,b show the SEM images of the nanofingers before and after the collapsing process. A group of four nanofingers formed a stable tetramer nanostructure through capillary forces. Figure 2c is the TEM image of the nanogap between the two nanoparticles. A 4 nm gap is clearly shown in the middle of the two Au nanoparticles, which is twice the thickness of the ALD-coated TiO_2_ film. The composition of the collapsed nanofingers were analyzed using EDS, and the results are shown in Figure 2d–f. The nanogap which exists in the Au mapping disappears in the Ti and O mappings, indicating that the TiO_2_ film serves as the spacer to define the 4 nm gaps between the two nanoparticles.

It is crucial to optimize the gap size as it can significantly impact the intensity of the electric field enhancement. Based on a classical electromagnetic model, an increment in the field enhancement at the hot spot [44] and a redshift of the plasmon resonance [45] can be observed as the distance between the two nanoparticles decreases. However, further theoretical studies have shown that quantum mechanical effects should be considered as the gap size reaches to a few nanometers [36,37,38,46,47,48]. As two metal nanoparticles approach each other, stronger tunneling through the metal–dielectric–metal interface can be expected and therefore limit the field enhancement [41,42,43,49]. Based on energy band diagrams, the tunneling barrier height is equal to the difference in the Fermi energy of gold (5.1 eV) and the electron affinity (EA) of the dielectric [50]. In our case, the relatively large EA (=4.21 eV) of TiO_2_ led to a low barrier height of 0.89 eV. In other words, the electric field enhancement will be limited when the thickness of the TiO_2_ film is smaller than the threshold due to the strong tunneling effect. Moreover, the volume with a relatively high-field enhancement factor is tens of cubic nanometers, and an ultrathin TiO_2_ film cannot cover the entire hot spot, resulting in a low energy conversion rate. ALD is an ideal technique for our process as it can deposit a subnanometer-level thin dielectric film and precisely control the thickness. According to our previous work, a 4 nm gap size can provide the strongest field enhancement factor at the hot spots [26].

**Figure 3:**
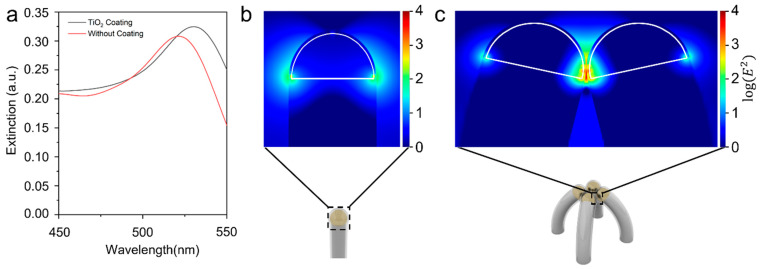
(**a**) Extinction spectra of nanofingers without and with 2 nm TiO_2_ coating. (**b**) Simulated electric field distribution for uncollapsed nanofingers. (**c**) Simulated electric field distribution for collapsed nanofingers.

To show that the collapsible nanofingers can produce strong gap plasmon and thus local field enhancement, we performed an FDTD numerical simulation to study the field distribution near the nanofinger surface. Figure 3a shows that the extinction spectrum of the nanofinger coated with a 2 nm TiO_2_ film has a peak at ~530 nm. The optical response of the nanofinger is not significantly affected by the dielectric coating layer, except for a slight spectral redshift of the resonant peaks, which corresponds to the refractive index variation introduced by the TiO_2_. In the collapsed nanofingers, the bonding dimer plasmon (BDP), originating from the hybridization of the dipolar plasmon modes of the single nanoparticles, and the charge transfer plasmon (CTP), referring to the electron tunneling between the nanoparticles, are the two competing modes [36,51]. An optimal gap size, which is 4 nm in our work, can provide the strongest field enhancement. Figure 3c shows the field distribution around the gap of the collapsed nanofingers. The field enhancement at the hot spot is much stronger compared to the uncollapsed nanofinger that is shown in Figure 3b. This is because the collapsed nanofingers can form Au-TiO_2_-Au interfaces and produce gap plasmon at the interfaces. The gap plasmon can produce a much higher field enhancement compared to the surface plasmon produced by single nanoparticles.

**Figure 4:**
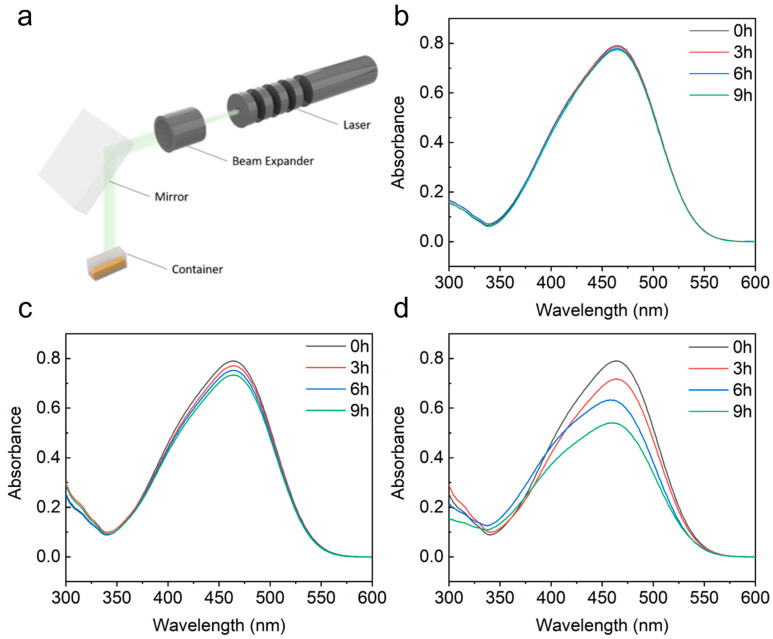
(**a**) Schematic of photocatalysis experiment set up. (**b**–**d**) UV–Vis spectra of MO aqueous solution before and after 3 h, 6 h, 9 h visible light exposure using 2 nm TiO_2_ film, isolated nanofingers and collapsed nanofingers as photocatalysts.

The photocatalytic activity of our plasmonic photocatalyst was evaluated using the photo-degradation of methyl orange (MO) under visible light irradiation. The experiment set up is shown in Figure 4a. The photocatalyst and MO solution were added into a transparent reaction vessel. A 532 nm laser beam was used as the light source. The whole set up was covered by a black curtain to exclude other light. In the control experiments, a TiO_2_ thin film (a silicon substrate-coated 2 nm TiO_2_ film using ALD) and uncollapsed nanofingers were used as photocatalysts, respectively. Figure 4b–d show the MO absorption spectra at different irradiation times using these three different samples as the photocatalysts. As there was no enhancement in the bare TiO_2_, only 1.5% of the MO was degraded after a 9 h reaction. For the uncollapsed nanofingers, the plasmon produced by the Au nanoparticles that are capped on the nanofingers can generate hot carriers to promote the photocatalytic activity of TiO_2_. As a result, the MO absorbance was observed to drop by 6.5% after a 9 h reaction, and there was a four-fold photo-degradation rate improvement compared with the bare TiO_2_. A 33% reduction in the MO absorbance was observed when the collapsed nanofingers were used as the photocatalyst. This over-twenty-fold improvement is contributed to the much stronger gap plasmon produced at the gap between the two nanoparticles. The kinetic model of the photocatalytic degradation of MO can be described using the Langmuir–Hinshelwood pseudo first-order kinetics equation, which is expressed as:

$$\ln\frac{C_{0}}{C}=Kt$$

**Figure 5:**
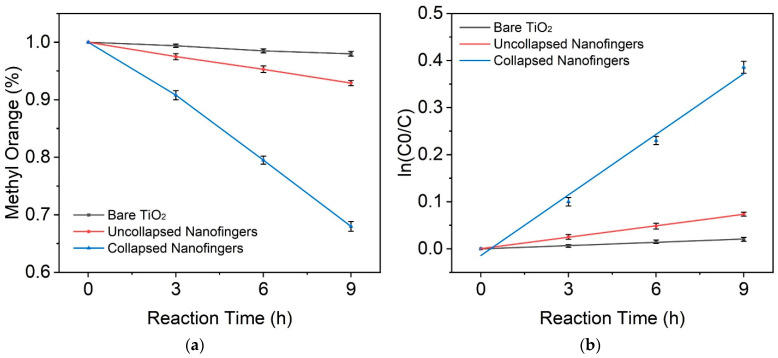
(**a**) Concentration of methyl orange dye at different reaction times. (**b**) Modeling of the photocatalytic kinetics of methyl orange according to the Langmuir–Hinshelwood model.

The variable $C_{0}$ and C in the equation are the initial and final concentration of MO dye, and K is the reaction rate constant. Figure 5 shows the concentration of MO dye at the different reaction times and the fitting result using the kinetics equation. For the photocatalytic degradation of MO using bare TiO_2_, uncollapsed nanofingers and collapsed nanofingers, the reaction rate was 0.00232, 0.00813 and 0.043. The photocatalytic degradation perfectly followed the pseudo first-order kinetic in all of the three cases.

**Figure 6:**
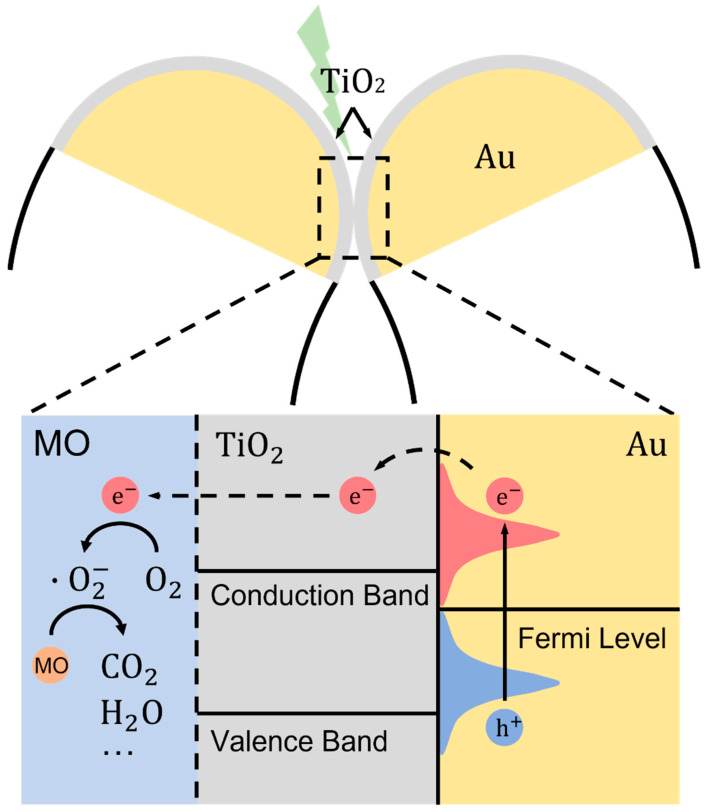
Illustration of the hot carrier-driven photocatalytic degradation of MO on collapsed nanofingers.

Plasmon-induced photocatalytic activity can originate from the interaction of Au nanoparticles with TiO_2_ film. The hot carrier injection mechanism on the collapsed nanofingers is illustrated in Figure 6. The photoexcitation of the AuNPs generates plasmonic resonance near the surface, resulting in a nonthermal distribution of hot electrons above the Au Fermi energy and hot holes below the Fermi energy. As the population of the hot electrons is proportional to the energy coupled into the AuNPs, it can be inferred that the total amount of hot electrons scales with the square of the localized field enhancement [26]. The excitation light source for our plasmonic structure will induce the strongest plasmonic resonance and hence the largest local field enhancement, which can be verified by previously reported UV-Vis absorption spectra and FDTD simulations. Under 532 nm excitations (photon energy: 2.3 eV), the decay of plasmon resonances resulted in energetic hot electron distributions with energy up to 2.3 eV above the Fermi level of Au. Importantly, the relatively low edge of the TiO_2_ conduction band (CB ~4.21 eV vs. vacuum) led to a low Schottky barrier (0.89 eV) across the Au-TiO_2_ interface for the hot electron injection. In this case, most of the population of the hot electrons gained sufficient energy to get injected into the CB of TiO_2_, followed by the energetic favorable transfer process into the $\pi^{*}$ orbital of the molecular O_2_ adsorbed on the TiO_2_ surfaces to form $O_{2}^{-}$ that can be further transformed into  OH [6,52,53,54]. The resulting radicals are very strong oxidizing agents and can oxidize aromatic organic compounds into small molecules, such as CO_2_ and H_2_O [6]. A recent study has shown that lasers can activate singlet oxygen species to form radicals with the help of the scattering effect of semiconductor particles or the lens effect of the solution [55]. As the samples are coated by a TiO_2_ thin film and the surface area of the solution is much larger than the laser beam size, this pathway does not contribute to the formation of radicals in this experiment. In that sense, the total reaction rate in our experiments should be guided by ${\left|E\right|}^{2}$. Based on the FDTD simulations, the photocatalytic enhancement factor can be estimated using the following equations:

$$\mathrm{EF}=\frac{\oint_{{\mathrm{volume}\;\mathrm{of}\;\mathrm{TiO}}_{2}}^{\;}{\left|E\right|}^{2}}{\oint_{{\mathrm{volume}\;\mathrm{of}\;\mathrm{TiO}}_{2}}^{\;}{\left|E_{0}\right|}^{2}}$$

where E is the electric filed inside TiO_2_, and E_0_ is the electric field of the incident light. As the hot electron diffusion length inside TiO_2_ is much larger than 2 nm, we integrated over the whole volume of the TiO_2_ film. Upon performing this integral, we obtained the theoretical plasmon resonant enhancement factors for the uncollapsed and collapsed nanofingers as 9.8 and 21.3, respectively, which are consistent with our experiment observation ($\frac{0.00813}{0.00232}=3.5$ for uncollapsed nanofingers, and $\frac{0.043}{0.00232}=18.5$ for collapsed nanofingers). Therefore, our optimized gap plasmonic nanostructures, with field enhancement approaching theoretical limits [26,27,36,38,51], offer the most substantial amount of hot electrons with an energetically favorable injection and thus promote the photocatalytic activity of TiO_2_ under visible light.

## 4. Conclusions

In summary, we have demonstrated that large-area gap plasmon nanostructures can be used as plasmonic photocatalysts with high activity under visible light. Based on our developed method of fabricating large-area collapsible nanofingers, we can combine AuNPs and thin TiO_2_ films to create a novel plasmonic photocatalyst. The AuNP pairs can produce ultra-strong plasmonic resonance between the two particles, and the TiO_2_ film is exactly placed at the hot spots to utilize the harvested energy to accelerate the chemical reaction. The FDTD simulations of this nanostructure show that the enhanced photocatalytic activity is due to the large enhancement of the local electric field at the hot spots, which promote the formation of hot carriers and hence increase the photodecomposition rate of methyl orange. This nanostructure provides a good platform for a deeper understanding of hot carrier-driven photocatalysis and paves the way for the future design of plasmonic photocatalysts.

## Data Availability

Not applicable.

## Supplementary Materials

The following supporting information can be downloaded at: https://www.mdpi.com/article/10.3390/nano12213730/s1, Figure S1: Fabrication procedure of collapsible nanofingers.
