# Supplementary material for: Hot Electron-Driven Photocatalysis Using Sub-5 nm Gap Plasmonic Nanofinger Arrays
Source: Nanomaterials (Basel). 2022 Oct 24;12(21):3730. doi: 10.3390/nano12213730 (PMC9655529; doi:10.3390/nano12213730)
Supplement: Supplementary file 1 [file nanomaterials-12-03730-s001.zip › nanomaterials-1944132-supplementary.pdf]

Supplementary Materials

Hot Electron Driven Photocatalysis using Sub-5 nm Gap Plasmonic Nanofinger Arrays

*Yunxiang Wang[1], Buyun Chen[1], Deming Meng[1], Boxiang Song[2], Zerui Liu[1], Pan Hu[1], Hao Yang[1], Tse-Hsien Ou[1], Fanxin Liu[3], Halton Pi[4], Irene Pi[5], Isleen Pi[5] and Wei Wu[1]**

[1]Ming Hsieh Department of Electrical and Computer Engineering, University of Southern California, Los Angeles, CA 90089, USA.

[2]Huazhong University of Science and Technology, Wuhan National Laboratory for Optoelectronics, Wuhan 430074, China

[3]Department of Applied Physics, Zhejiang University of Technology, Hangzhou 310023, China

[4]Biophysics, Johns Hopkins University, Baltimore, MD 21218, USA

[5]Torrey Pines High School, San Diego, CA 92130, USA

Corresponding author. E-mail: wu.w@usc.edu

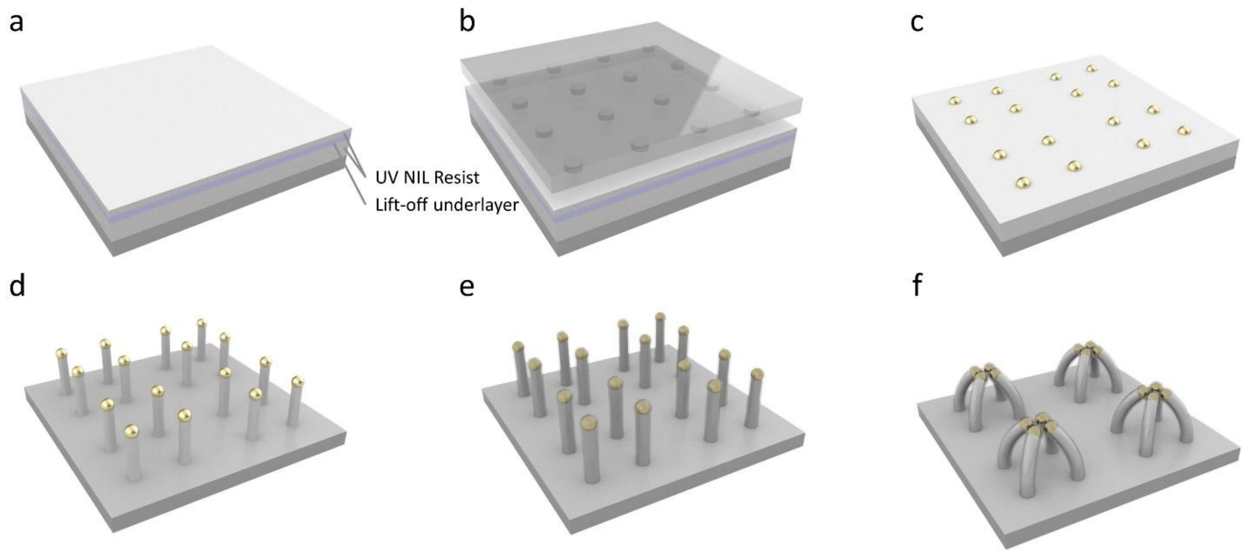

Figure S1. Fabrication procedure of collapsible nanofingers. a) Spin-coat 600 nm UV NIL resist, 100nm lift-off layer and 100nm UV NIL resist on the substrate. b) Perform UV curable nanoimprint and then etch residual layer and lift-off layer. c) Deposit 50 nm Au followed by lift-off process to form Au nanoparticle array onto the thick UV imprint resist layer. d) Perform RIE to obtain nanofinger array. e) Deposit 2 nm TiO_2 thin film on the nanofinger array using ALD. f) Soak fabricated sample into ethanol solution and then air-dry at room temperature to get collapsed nanofinger array.
